# Video Foreground Detection Algorithm Based on Fast Principal Component Pursuit and Motion Saliency

**DOI:** 10.1155/2019/4769185

**Published:** 2019-02-03

**Authors:** Rui Chen, Ying Tong, Jie Yang, Minghu Wu

**Affiliations:** ^1^College of Communication Engineering, Nanjing Institute of Technology, Nanjing 211167, China; ^2^School of Electrical and Electronic Engineering, Hubei University of Technology, Wuhan 430068, China

## Abstract

Aiming at the shortcoming of being unsuitable for dynamic background and high computational complexity of the existing RPCA- (robust principal component analysis-) based block-sparse moving object detection method, this paper proposes a two-stage foreground detection framework based on motion saliency for video sequence. At the first stage, the observed image sequence is regarded as the sum of a low-rank background matrix and a sparse outlier matrix, and then the decomposition is solved by the RPCA method via fast PCP (principal component pursuit). At the second stage, the sparse foreground blocks are obtained according to the spectral residuals and the spatial correlation of the foreground region. Finally, the block-sparse RPCA algorithm through fast PCP is used to estimate foreground areas dynamically and to reconstruct the foreground objects. Extensive experiments demonstrate that our method can exclude the interference of background motion and change, simultaneously improving the detection rate of small targets.

## 1. Introduction

Moving object detection plays a critically important role in computer vision systems, such as intelligent video surveillance, behavior analysis, and so on. The aim of detection is to separate the moving objects called “foreground” from the static information called “foreground” in video sequences. The effectiveness of moving object detection methods is very important for the postprocessing of object tracking, target classification, behavior understanding, and so on. Among all kinds of moving object detection methods, the background subtraction is considered as the most effective method to extract the foreground object. This method compares the pixels of an image with a background model and considers those that differ from the background model as moving objects. So, the key of this method is the effective background modeling, which directly affects the accuracy and robustness of the whole detection system.

There are two main types of background modeling. (1) Pixel-level-based background model, including the Mixture Of Gaussian (MOG) model [1], Bayes model [2], Markov random field (MRF) model [3], Code Book mode [4], and pixel sampling (ViBe) model [5]. These methods model the background for each pixel independently, not considering the correlation and structural characteristics of pixels. So, the detection results are not ideal in complex and varied video scenes and these methods are not robust against global variations such as illumination changes. (2) Image-level-based background model. This model is based on subspace estimation, i.e., sparse representation and rank minimization. Robust Principal Component Analysis (RPCA) decomposes a data matrix *D* in two components such that *D* = *L* + *S*, where *L* is a low-rank matrix and *S* is a sparse matrix. Candes et al. proposed a convex optimization to address RPCA problem and showed results with the *L* + *S* decomposition model in computer vision applications like background modeling [6]. This RPCA formulation suffices in applications where the information of interest is in matrix *L*, such as image denoising and image alignment. Applying it in applications in which there is also information of interest in the sparse matrix *S* (such as background/foreground separation) results that outliers contains both the information of interest (moving objects that is considered as sparse) and the noise [7]. But, Candes's work has aroused a resurgence of interest in RPCA and extensions in computer vision.

**Figure 1:**
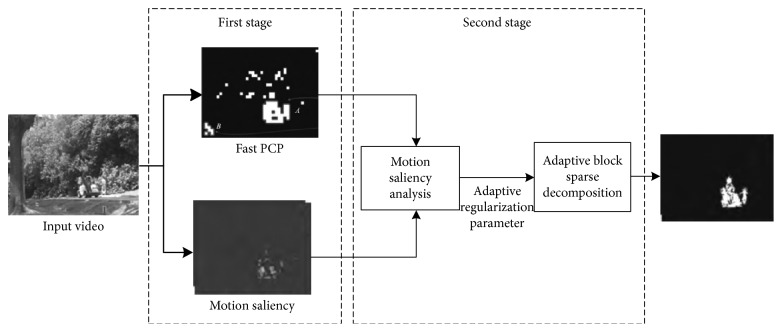
The two-stage framework of our proposed foreground detection algorithm.

Recently, many approaches have been developed and can be classified into [8] (1) RPCA via Principal Component Pursuit (PCP), noted as PCP-RPCA; (2) RPCA via outlier pursuit; (3) RPCA via iteratively reweighted least squares; (4) Bayesian RPCA; and (5) approximated RPCA. In this paper, we focus on PCP-RPCA, in which the background matrix is the constraint using kernel norm, and foreground matrix is the constraint using *l*_1_-norm. The PCP-RPCA method does not need a large number of initial training to get the background model and does not need to set many output parameters like the mixed Gauss model. The background estimation and the foreground detection can be implemented at the same time using matrix decomposition. Although the PCP-RPCA method is becoming a research hotspot, it still needs to be improved in the following aspects: (1) the high computation complexity of solving matrix equation; (2) the parameters in PCP method are generally fixed leading to the greater impact of the dynamic background on the foreground extraction. In this paper, we propose a two-stage foreground detection framework based on motion saliency for video sequences, which is shown in Figure 1. Motion saliency is introduced to address the parameter setting issue in dynamic background videos and to tune regularization parameters adaptively. At the first stage, the observed image sequence is regarded as the sum of a low-rank background matrix and a sparse outlier matrix, and then the decomposition is solved by the RPCA method via fast PCP. Motion block candidates are obtained. At the second stage, a fast motion saliency detection method based on spectral residual is applied to these candidates, and the adapt block sparsity decomposition is used to detect the foreground. Finally, the block-sparse RPCA algorithm through fast PCP is used to estimate foreground areas dynamically and to reconstruct the foreground objects.

## 2. Related Works

Principal Component Analysis (PCA) is a fundamental technique to find the principal components' space with the smallest dimension that spans a given dataset. Let *D* ∈ *R* ^*m*×*n*^ be a high-dimensional matrix (i.e., the observed matrix). For video sequences, *D* is composed of multiple video frames rearranged through column rearrangement. Each column of *D* corresponds to one frame of the original video. Matrix *D* can be represented by

(1) $$\begin{array}{c} D=L+N, \end{array}$$

where *L* is the low-rank component of matrix *D* and *N* is the perturbation matrix. The classic PCA method seeks the best rank-*k* approximation with complexity *O*(*kmn*) [9] and can be described as finding the optimal rank to estimate *L* by

(2) $$\begin{array}{c} \arg\;\;\min\;\left\|D-L\right\|,\\ \text{s}.\text{t}.\;\text{rank}\left(L\right)\leq k, \end{array}$$

where ‖·‖ represents the *l*_2_-norm, i.e., the maximum singular value of *D* and rank (*L*) is the rank of matrix *L*. When the noise is small, Equation (1) can be solved by the Singular Value Decomposition (SVD) method, and its optimal solution is obtained when the nonzero elements in sparse matrix L are independent and identically distributed.

It is known that PCA is sensitive to outliers and then RPCA is proposed. RPCA solves the deficiency of the valid data loss in the dimensionality reduction process of PCA, so it is widely used in the moving object detection for video sequences. The basic idea of these RCPA models is suppose a matrix *D* can be decomposed into a low-rank matrix *L* and a sparse matrix *S*, then *L* and *S* can be obtained by some mathematical method. Ideally, an image can be expressed as a superposition of a dynamic foreground and a static background. Similarly, a column-vectorized (i.e., a column in the matrix is a video frame) video sequence can be decomposed into a low-rank matrix *L* and a sparse matrix *S*. The background matrix is considered to have a low-rank characteristic because of the strong correlation. The foreground matrix has a sparse characteristic.

Similar to (1), Candès et al. [6] pointed out that matrix *D* can be decomposed as

(3) $$\begin{array}{c} D=L+S, \end{array}$$

where *S* is the sparse component of matrix *D*. Then (2) can be rewritten as

(4) $$\begin{array}{c} \underset{L,S}{\arg\;\;\min}\;\text{rank}\left(L\right)+\lambda {\left\|S\right\|}_{0},\\ \text{s}.\text{t}.\;D=L+S, \end{array}$$

*λ* is the regular parameter, ‖*S*‖_0_ represents the *l*_0_-norm of matrix *S*, i.e., the number of the nonzero elements of matrix *S*. Furthermore, (4) can be translated into convex optimization as

(5) $$\begin{array}{c} \underset{L,S}{\arg\;\;\min}\;{\left\|L\right\|}_{*}+\lambda {\left\|S\right\|}_{1},\\ \text{s}.\text{t}.\;D=L+S, \end{array}$$

‖*L*‖_*∗*_ is the kernel function of matrix *L* and represents the sum of the singular values of *L*, i.e., ‖*L*‖_*∗*_=∑_*i*=1_ *σ*_*i*_(*L*). ‖*S*‖_1_ is the *l*_1_-norm of matrix *S* and represents the sum of the absolute values of all elements in *S*, i.e., ${\left\|S\right\|}_{1}=\underset{j}{\max}\left(\sum_{i=1}^{m}\left|a_{i,j}\right|\right)\left(j=1,2,\ldots ,n\right)$.

Equation (5) is the optimization objective function of the RPCA method. Under the condition that the number of rows and columns of *L* is unknown, and the number and position of nonzero elements in *S* is also unknown, Candes et al prove that (5) can be solved by using the PCP algorithm. So the low-rank component and sparse component can be completely or effectively reconstructed.

To solve RPCA problem, ALM (Augmented Lagrangian Multiplier) is proposed, and the ALM operates on the augmented Lagrangian, which is described as follows:

(6) $$\begin{array}{c} \underset{L,S,Y}{\arg\min}{\left\|L\right\|}_{*}+\lambda {\left\|S\right\|}_{1}+\langle Y,D-L-S\rangle +\frac{\mu }{2}{\left\|D-L-S\right\|}_{F}, \end{array}$$

where *λ* is the augmented Lagrange operator, and *μ* is the positive penalty parameter. Based on it, many ALM's variants have been proposed. Cai et al. proposed IT (Iterative Thresholding) RPCA [9], Patrinos et al. exploited the APG (Accelerated Proximal Gradient) algorithm to solve the RPCA model [10], and Lin et al. presented the IALM- (Inexact Augmented Lagrange Multiplier-) based RPCA [11]. Since the core computation of these algorithms depends on SVD computations, the time complexity is *O*[min(nm^2^, mn^2^)] which is unacceptable for some specific real-time applications. Addressing on high computation cost of multiple SVDs, some approaches have been proposed [12–14]. In [12], the authors proposed the FRPCA method-integrated abstract information of the offline-recovered low-rank of the input data rather than including the entire dataset, decreased the time complexity while sacrificing little accuracy. In [13], the authors proposed a fast trifactorization method to approximate the nuclear norm minimization problem and mitigate the computation cost of performing SVDs. Yang [14] proposed an ALM's variant called the ADMM (Alternating Direction Methods of Multipliers) method for RPCA by smoothing the nonsmooth terms in objective function and the fast operator to improve the convergence rate.

To achieve speedup, Rahmani [15] presented a noniterative RPCA algorithm, named Coherence Pursuit (CoP). CoP sets an outlier apart from an inlier by comparing their coherence with the rest of the data points. The mutual coherences are computed by forming the Gram matrix of the normalized data points. Subsequently, the sought subspace is recovered from the span of the subset of the data points that exhibit strong coherence with the rest of the data. Ebadi [16] presented an approximated RPCA framework for recovery of a set of linearly correlated images seeking an optimal solution for decomposing a batch of realistic unaligned and corrupted images as the sum of a low-rank and a sparse corruption matrix, while simultaneously aligning the images according to the optimal image transformations. In [17], the authors extended the AltProj [18] method and presented a factorization-based model of RPCA, which has a complexity of *O*(*kmn*), where *k* is an upper bound of the true rank.

Moreover, some research studies addressed on dynamic RPCA which assume that the subspace from which the true data are generated can change slowly with time. Relying on the recently studied recursive projected compressive sensing (ReProCS) [19] framework for solving dynamic RPCA problems, Narayanamurthy [20] proposed a recursive projected compressed sensing based algorithm with nearly optimal memory complexity and is almost as fast as vanilla SVD. The overview of dynamic RPCA solutions are described in [21] and it can be referred.

Addressing the limitation of real-time applications, it would be more useful to quickly estimate the low-rank matrix and the sparse matrix in an incremental way for each new frame, rather than as a batch. Rodríguez [22] proposed an incremental PCP algorithm that can process one video frame at a time, obtaining similar results to standard batch PCP algorithms at low-memory footprint and real-time processing speed. Qiu [23] proposed a real-time robust PCP solution that automatically handles correlated sparse outliers which still require the singular vectors of the low-rank part to be spread out, but does not require i.i.d. of either the sparse part or the low-rank part.

## 3. Foreground Detection Based on Decomposition of Low-Rank and Sparsity Matrices

### 3.1. Fast PCP Algorithm

Because the calculation of RPCA-PCP model is a costly optimization problem and there is urgently to deal with large amounts of data in real time, we employ a fast PCP algorithm. Most of variants of (5) are constructed by changing the penalty constraint and vice-versa. One of the variants is

(7) $$\begin{array}{c} \underset{L,S}{\arg\;\;\min}\;\frac{1}{2}{\left\|L+S-D\right\|}_{F}+\lambda {\left\|S\right\|}_{1},\\ \text{s}.\text{t}.\;{\left\|L\right\|}_{*}\leq t. \end{array}$$

Noting that we are interested in solutions under the constraint ‖*L*‖_*∗*_ ≤ *t*, we get the equality of (7) as

(8) $$\begin{array}{c} \underset{L,S}{\arg\;\;\min}\;\frac{1}{2}{\left\|L+S-D\right\|}_{F}+\lambda {\left\|S\right\|}_{1},\\ \text{s}.\text{t}.\;\text{rank}\left(L\right)=t. \end{array}$$

The *L* subproblem corresponds to an NNR (Nuclear Norm Regularization) problem [24] which can be efficiently solved in [25, 26]. In [9], the authors have implemented this algorithm and proposed a solution method.

The alternating minimization can be used to solve (8) as

(9) $$\begin{array}{c} L_{k+1}=\underset{L}{\arg\;\;\min}\;{\left\|L+S_{k}-D\right\|}_{F}, \end{array}$$

(10) $$\begin{array}{c} S_{k+1}=\underset{S}{\arg\;\;\min}\;{\left\|L_{k+1}+S-D\right\|}_{F}+\lambda {\left\|S\right\|}_{1}. \end{array}$$

Equation (9) can be solved by computing a partial SVD of *D* − *S*_*k*_ with *t* components. Since the matrix *L* typically has very low rank, we can simplify the procedure to estimate an upper bound for *t* in (9) as

1. input video *D*, initialize parameter: *S*_1_ = 0, rank = 1 (the initial rank)
2. while not converge do
3. solve *L*_*k*+1_ with rank = *t* and preserve singular values to *v*
4. calculate (*v*_rank_/∑_*k*=1_^rank^ *v*_*k*_)
5. if (*v*_rank_/∑_*k*=1_^rank^ *v*_*k*_) > *τ* then  rank++
6. solve *S*_*k*+1_
7. end while
8. output: *L*, *S*

This method only needs a very low number of iterations, and the memory requirement is not large. The experimental results show that this method can meet the application of real-time video processing with an acceptable detection effect.

### 3.2. Performance of Fast PCP Algorithm

In order to verify the effectiveness of the fast PCP algorithm, we compare it with the inexact ALM algorithm [27] and the nonsmooth augmented Lagrangian algorithm noted as NSAL [28]. The test video is a 400-frame traffic video sequence at 15 fps. The original size of each frame has 640 ∗ 480 pixels, and each pixel value is normalized to 0∼1. We also consider a reduced size version of 320 ∗ 240 pixel per frame.

The experimental results are presented from the following two aspects: (1) the time consumption on a given number of iterations; (2) the reconstruction quality ‖*S*_*GT*_ − *S*_*k*_‖_1_/*N*, where *S*_GT_ represents the ground truth sparse video approximation, *S*_*k*_ represents the sparse video approximation at the *k*-th outer loop, and *N* represents the number of pixel per frame used as a normalization factor.

**Figure 2:**
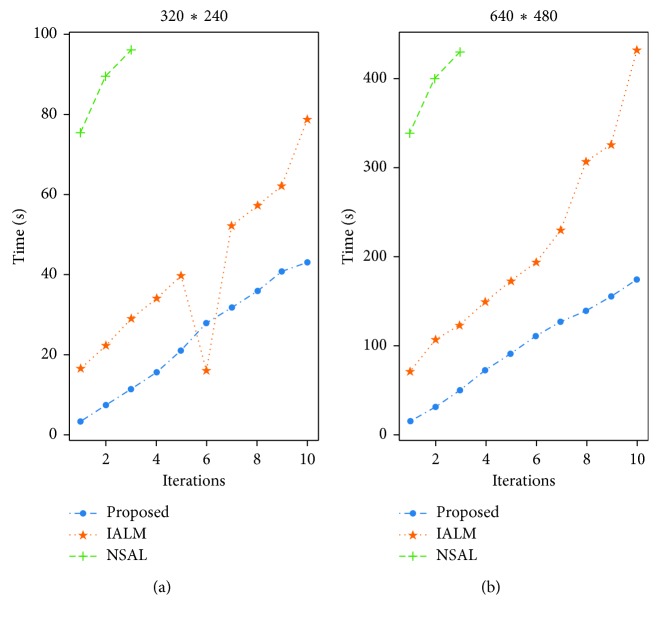
Computational performance comparison of fast PCP, IALM, and NSA algorithms.

The computational performance is shown in Figure 2. We compare our proposed algorithm with the inexact ALM and NSAL for the video background removal problem using the abovementioned 400-frame (640 ∗ 480) video sequence. At the same given iteration number, our proposed algorithm is faster than the inexact ALM about 2 times per iteration and much faster than NSAL about 8 times per iteration. We only report the performance for the first ten loops due to space constraints for all the considered algorithms (the NSA algorithm was only run from one up to three outer loops because the improvement of the solution is only incremental after the third loop).

**Figure 3:**
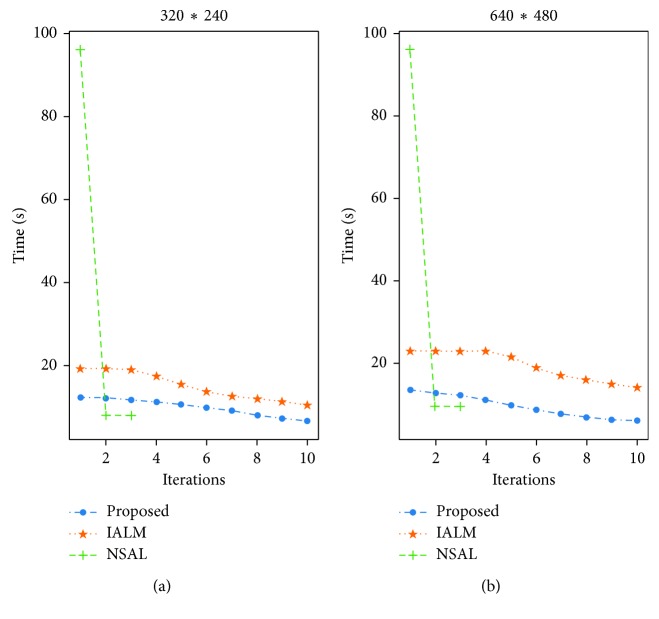
Reconstruction quality performance comparison of fast PCP, IALM, and NSA algorithms.

Furthermore, the reconstruction quality is improved as shown in Figure 3. This is due to the fact that the inexact ALM algorithm requires a certain number of iterations to achieve better quality. As we can see from Figure 3, it takes at least 11 iterations to achieve the result of the first iteration of the fast PCP algorithm. The NSAL needs a large amount of time to finish its second iteration. Therefore, fast PCP algorithm can process video sequences in real time and has a good foreground detection effect. It is very suitable to be used as the first step of video foreground detection.

## 4. Adaptive Block Sparse Decomposition with Spectral Residual Model

After completing the image's low-rank and structured sparse decomposition, we obtained many motion candidate blocks. However, due to the difficulty of parameter selection in matrix decomposition, these motion candidate blocks contain both real foreground and background motion. In this section, motion saliency analysis technique is applied to discriminate which candidate blocks belong to background motion and which belong to real foreground motion.

On the contrary, in the matrix decomposition method, it is difficult to choose a single parameter to adapt to all scenarios. Simply speaking, because the parameter *λ* in (4) controls the ratio of background and foreground after matrix decomposition, then for foreground detection tasks, it is hoped that a smaller value *λ* will lead to a complete foreground target; for background estimation tasks, it is also hoped that the value *λ* will be smaller, so there will be no foreground shadows in the recovered background. Considering the importance of getting a complete target, a small and fixed value of *λ* is often used in previous algorithms, but this global and unified parameter setting makes it difficult to remove the disturbance of complex and changeable background motion.

### 4.1. Spectral Residual Model

The main purpose of saliency detection in video sequences is to find the salient motion regions from the background, which is different from the traditional saliency detection in images. The main idea is to roughly remove the redundant part of a volume data (the static part of temporal slices) and keep the salient motion regions. Accurate and efficient positioning of significant motion targets is a very important preprocessing process in many video understanding applications, but it has always been a challenging problem because today's video or 3D stereodata contain a variety of background movements.

A fast motion saliency detection algorithm based on temporal spectral residual was proposed to solve this problem [29]. The principle of this method is for the significant motion, the moving target area contains different signals on the cross section of the time axis, while the background area contains redundant information. The main idea of the algorithm is to extract significant information on the cross section by using a two-dimensional image saliency detection method, i.e., spectral residual (SR) method. After that, the majority voting strategy is introduced to obtain the final reliable results. Because this method only involves Fourier spectrum analysis, it has high computational efficiency. This algorithm can extract reliable motion areas and does not need initial marks or any training data.

Spectral residual (SR) is a saliency detection algorithm on 2D images, and its main idea is to roughly remove the redundant part of a volume data (the static part of temporal slices) and keep the salient motion regions. The algorithm is different from the methods of summarizing the attributes and characteristics of the target object and mainly depends on the difference of the power spectrum of the logarithm of the image and focuses on the attributes of the image background. It focuses on the observation that log spectra of different images share similar trends, though each containing statistical singularities. The similarities imply redundancies. If the similarities are removed, the remaining singularity should be the visual saliency of an image. The algorithm only needs Fourier transform on an image, so it is computationally efficient. The SR algorithm can be described as follows.

Given an original image *I*(*x*) and its Fourier spectrum *f*: *A*(*f*)=|*F*[*I*(*x*)]| and *P*(*f*)=*φ*(*F*[*I*(*x*)]), the log spectrum representation of *f* is

(11) $$\begin{array}{c} L\left(f\right)=\log\;\;A\left(f\right). \end{array}$$

Since the log curve satisfies the local linear condition, an approximated solution of *L*(*f*) with a local average filter *h*_*n*_(*f*) can be obtained by $\bar{L\left(f\right)}=h_{n}\left(f\right)*L\left(f\right)$. The spectral residual *R*(*f*) between *L*(*f*) and $\bar{L\left(f\right)}$ is calculated by

(12) $$\begin{array}{c} R\left(f\right)=L\left(f\right)-\bar{L\left(f\right)}. \end{array}$$

**Figure 4:**
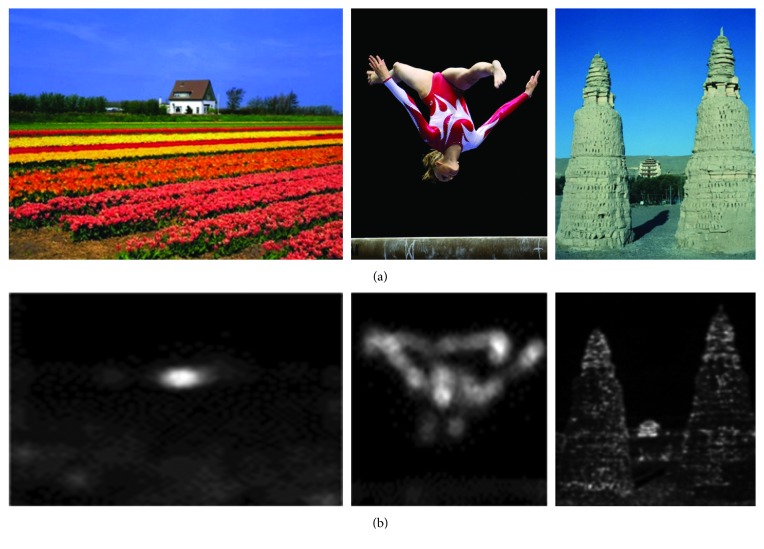
The saliency image of the SR algorithm. (a) Original image; (b) saliency results.

When the spectral residuals *R*(*f*) are transformed back to spatial domain, the high value pixels correspond to the salient regions. The IDFT (Inverse Discrete Fourier Transformation) of *R*(*f*) and *P*(*f*) is

(13) $$\begin{array}{c} S\left(x\right)={\left|F^{-1}\left[\exp\left\{R\left(f\right)+iP\left(f\right)\right\}\right]\right|}^{2}, \end{array}$$

where *S*(*x*) represents the saliency of each pixel in the initial image, i.e., the saliency map. Some examples of saliency map of the SR algorithm are shown in Figure 4.

### 4.2. Fast Saliency Motion Detection Based on Temporal Spectral Residual

The SR algorithm has been successfully applied to 2D natural scene images. But it cannot be directly applied to motion saliency in video sequences or 3D volume because the saliency information of the motion is essentially different from the pixel intensity distribution.

**Figure 5:**
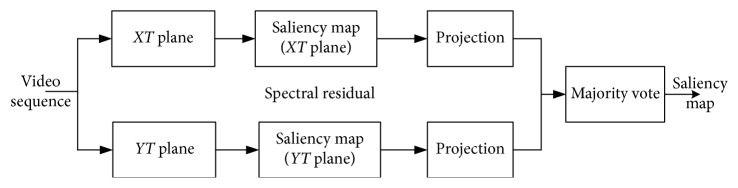
Temporal SR algorithm.

Generally, there are some basic observations in motion saliency detecting: (1) the region of background is usually greater than that of foreground; (2) foreground object motion is usually greater than background motion; (3) the background features of video are more unified than foreground. So, by analyzing the temporal slices in videos, the expected moving parts or obvious trajectories indicate the foreground moving objects. In this paper, we adopt the temporal SR algorithm in [29] to detect these obvious trajectories. The procedure of the algorithm is shown in Figure 5, where *T* is the temporal axis of the video sequence, *X* and *Y* are the axes of each frame, and then *XT* and *YT* represent the temporal slices, respectively. The original video sequences are split into the samples of temporal slices on the *XT* and *YT* planes to get the saliency map on *XT* and *YT* planes, respectively. Then the projections of the saliency maps of *XT* and *YT* in the original image sequences are obtained and the final saliency map is generated after a majority voting processing.

To detect the obvious trajectories of moving objects, the SR algorithm is applied on each temporal slice on the *XT* plane and the *YT* plane, respectively. Then the salient pixels are obtained. For example, the spectral residual of the temporal slice *I*_*XT*_ on *XT* plane can be calculated as

(14) $$\begin{array}{c} S_{XT_{j}}=\sigma \left(\text{SR}\left(I_{XT_{j}}\right)\right), \end{array}$$

where *j* is the index along *Y*-axis and SR(·) represents the SR algorithm used on slice image *I*_*XT*_*j*__. Because pixels with high energy value are more salient than those with lower values, a threshold function *σ*(*x*) is used in [29] to filter out pixels with low values as

(15) $$\begin{array}{c} \sigma \left(x\right)=\left\{\begin{array}{c} 1,\;\text{if}\;\;x>x_{\text{t}},\\ 0,\;\text{otherwise}, \end{array}\right. \end{array}$$

where parameter *x*_t_ is the threshold.

To further filter out the noisy pixels, evidences are collected on both temporal planes for the reason that the motions from background noises are usually random and orderless, and they rarely have distinct trajectories on both planes. If a salient pixel moves mainly along one direction, the majority voting is likely to discard it. The major salient regions still remain. A majority voting strategy is introduced to get the robustness of the results. Denoting *C* as the 3D volume that contains all saliency maps along the temporal plane, saliency majority voting is implemented on the *C*_*XT*_ and *C*_*YT*_ to obtain the final salient map by

(16) $$\begin{array}{c} C=C_{XT}\cdot C_{YT}. \end{array}$$

### 4.3. Block Sparse Decomposition with Adaptive Regularized Parameter Setting

**Figure 6:**
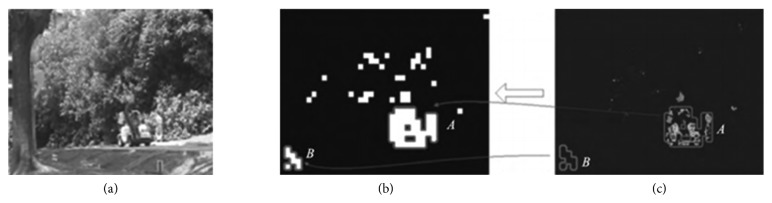
Motion saliency analysis. (a) One video frame. (b) Proposal blocks. (c) Motion saliency diagram.

For video sequences, there are some basic observations of a motion saliency detection task: (1) the region of foreground is often smaller than that of background; (2) background motion is often smaller than foreground object motion; (3) background has more regular patterns, even when dynamic background exists. Then, those blocks with low motion saliency will be considered as background and filtered out. As we can see from Figure 6(b), the candidate block *B* is detected as a moving foreground target by fast PCP algorithm. But it is not the real foreground because it is caused by the change of light (shadow) in the scene. To address this problem, the motion saliency at the block level is statistical analyzed to calculate the average motion saliency of all candidate regions. In the video sequence, those changes caused by illumination are not noticeable throughout the whole video. The average saliency value of candidate block *B* is significantly low in the entire time domain. Conversely, for the real foreground target block *A*, the motion saliency of each pixel in block *A* is very high, so the average saliency of block *A* is high. We can define a threshold *h* to remove those motion candidate areas whose saliency is below the threshold.

After motion saliency analysis, those proposal blocks with low average saliency are filtered out. If the location and size of each reserved candidate blocks are known, then we only need to calculate the motion saliency for foreground detection. The final foreground detection in the second stage is calculated by the block sparse RPCA [30] as (8). The regular parameter of block *i* is calculated by

(17) $$\begin{array}{c} \lambda_{i}=\frac{1}{10{\left(\max\left(m,n\right)\right)}^{1/2}}\frac{{\bar{M}}_{\min}}{\bar{M_{i}}}, \end{array}$$

where ${\bar{M}}_{\min}$ represents the lowest average saliency and $\bar{M_{i}}$ represents the average saliency of block *i*. Block sparse RPCA can be solved by the commonly used augmented Lagrangian method specified in [30].

## 5. Experimental Results

The detection performance is analyzed in detail in terms of the precision rate *p* and recall rate *F* as

(18) $$\begin{array}{c} P=\frac{P_{T}}{P_{T}+P_{F}},\\ R=\frac{P_{T}}{P_{T}+N_{F}}, \end{array}$$

where *P*_*T*_ is the number of pixels that are correctly judged as the foreground, *P*_*F*_ is the number of background pixels which is determined as the foreground, and *N*_*F*_ is the number of foreground pixels that are misjudged to be background. Taking these two factors into consideration, *F*-measure is the weighted harmonic mean of recall rate and precision rate [31]:

(19) $$\begin{array}{c} F=\frac{2P\times R}{P+R}. \end{array}$$

To evaluate the performance of our proposed algorithm, we compared 5 algorithms with dataset *Wallflower* and *I*2*R*. The tested videos include scenarios with little dynamic background interference test scenes (such as indoor moving people, vehicles on the road, and so on) and scenarios with dynamic background (such as elevators, branches, fountains, light change, and so on). We have chosen the most representative methods to compare with our proposed method: (1) DECOLOR method [32]; (2) MOG-RPCA method [33]; (3) PCP [30]; (4) FPCP [34]; and (5) R2PCP method [35].

**Figure 7:**
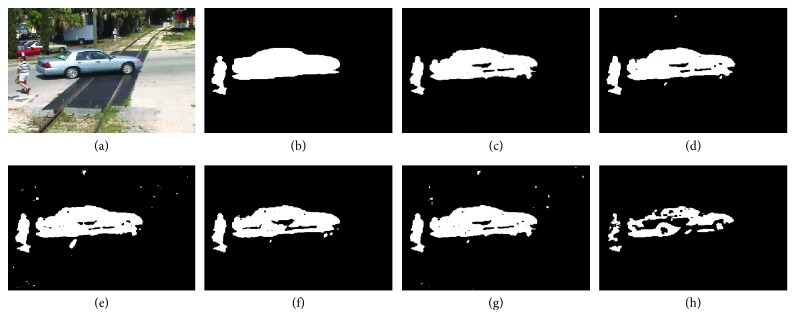
The results on BS sequence of Wallflower dataset. (a) Original query image; (b) the ground truth (GT) for the foreground; (c) our proposed algorithm; (d) DECOLOR; (e) MOG-RPCA; (f) PCP; (g) FPCP; (h) R2PCP.

**Figure 8 fig8:**
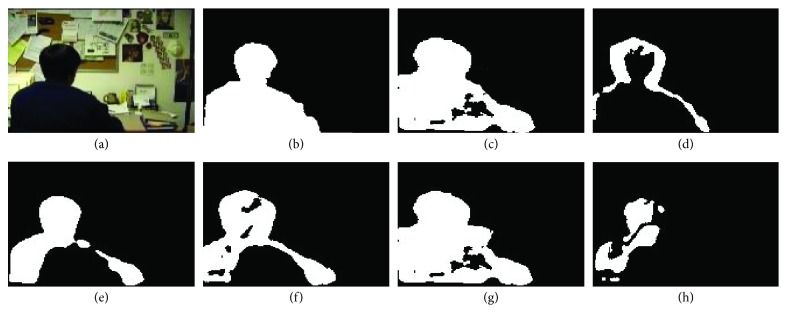
The results on FA sequence of Wallflower dataset. (a) Original query image; (b) ground truth (GT) for the foreground; (c) our proposed algorithm; (d) DECOLOR; (e) MOG-RPCA; (f) PCP; (g) FPCP; (h) R2PCP.

**Figure 9 fig9:**
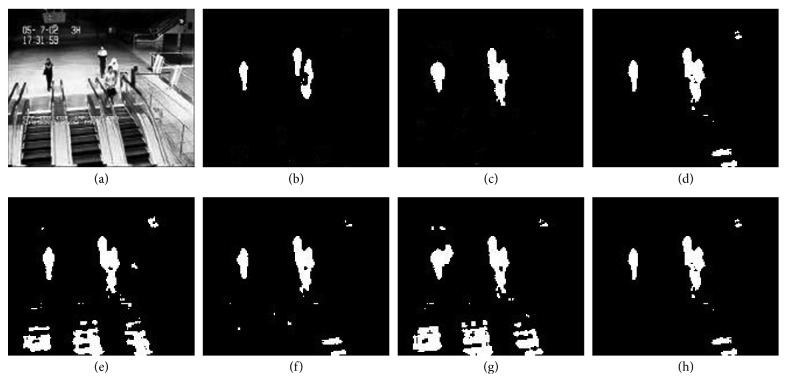
The results on ES sequence of I2R dataset. (a) Original query image; (b) the ground truth (GT) for the foreground; (c) our proposed algorithm; (d) DECOLOR; (e) MOG-RPCA; (f) PCP; (g) FPCP; (h) R2PCP.

**Figure 10:**
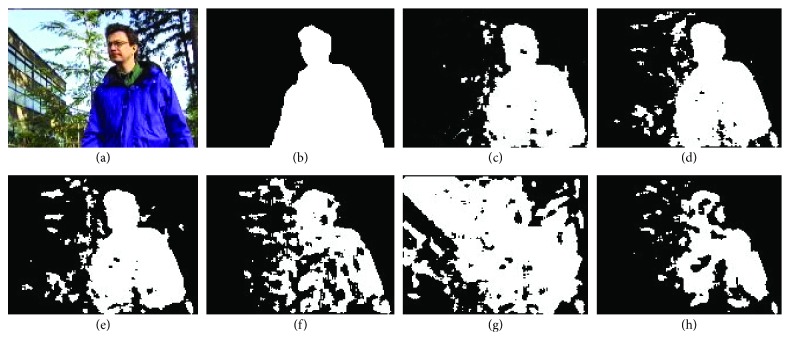
The results on WT sequence of Wallflower dataset. (a) Original query image; (b) the ground truth (GT) for the foreground; (c) our proposed algorithm; (d) DECOLOR; (e) MOG-RPCA; (f) PCP; (g) FPCP; (h) R2PCP.

The visual results on four video sequences are presented in Figures 7 [Fig fig8] [Fig fig9]–10. For *BS* sequence with little dynamic background interference, as shown in Figure 7, the results are visually acceptable except for R2PCP algorithm, and our proposed algorithm is closer to the GT (Ground Truth). For sequence “Foreground Aperture” (i.e., *FA* sequence shown in Figure 8), both our algorithm and the other four algorithms regard the part of the foreground target (the body of the sitting person) as the background result. The main reason is that the person in the video initially sleeps on the table and stays at rest for a long time, and when he is ready to get up and leave, most algorithms still assume that the part of the body that was initially lying on the table is the background. Our proposed algorithm overcomes this influence, and the detection effect is obviously better than other algorithms.

Figure 9 shows the foreground detection results of *ES* (Escalator) sequence in *I2R* database and *WT* (Waving Trees) sequence in Wallflower database. From the results shown in Figure 9, it can be seen that our proposed algorithm can eliminate the background interference of moving escalators and accurately detect the moving pedestrians. PCP, DECOLOR, and P2PCP algorithms can also detect pedestrians very well, but the escalator is not completely ruled out, and FPCP and MOG-RPCA algorithms regard the movement of escalator as the foreground. For *WT* sequence has dynamic motion (i.e., the trees behind people sway rapidly and continuously), the results of each algorithm are not good enough. Methods like FPCP and R2PCP fail to model the dynamic motion of the tree sways. DECOLOR method and our method have good results.

To further show the effectiveness of our method, we conducted quantitative analysis on *F*-measure scores shown in Table 1. For 7 sequences, we can see that our proposed algorithm has 5 highest *F*-measure scores and DECOLOR algorithm has 2 highest *F*-measure scores.

## 6. Conclusions

This paper proposes a two-stage foreground detection method based on motion saliency to overcome the interference of dynamic background. At first, a fast PCP algorithm is utilized to obtain the rough foreground and detect the proposal blocks. Then, the motion saliency index of each proposal block is detected by the SR algorithm, and the regular parameter of each proposal block can be determined according to the average saliency index. The final foreground targets are obtained by the RPCA algorithm based on block sparse decomposition. Compared with the existing foreground detection methods, our method has low computational complexity for real-time processing and overcomes the influence of dynamic background.

## Figures and Tables

**Table 1:** *F*-measure scores results on 7 sequences from Wallflower dataset.

Video sequence	*F*-measure score					
	Our proposed	DECOLOR	MOG-RPCA	PCP	FPCP	R2PCP
BS	**83.46**	80.55	82.78	80.42	79.56	78.88
CF	88.76	**90.55**	87.64	86.33	80.42	87.54
FA	**78.**89	22.37	54.37	56.74	70.65	33.14
GT	**90.**12	89.13	87.63	86.22	89.66	66.33
MO	**90.**87	88.53	89.29	87.87	84.77	76.37
WT	59.15	**60.22**	58.67	44.22	50.33	32.44
LS	**61.**57	34.84	44.89	50.78	58.47	41.83

## Data Availability

The [DATA TYPE] data used to support the findings of this study are included within the article.
